# Potential mechanism of RRM2 for promoting Cervical Cancer based on weighted gene co-expression network analysis

**DOI:** 10.7150/ijms.47356

**Published:** 2020-08-29

**Authors:** Jingtao Wang, Yuexiong Yi, Yurou Chen, Yao Xiong, Wei Zhang

**Affiliations:** Department of Obstetrics and Gynecology, Zhongnan Hospital of Wuhan University, Wuhan, Hubei 430071, P.R. China.

**Keywords:** Bioinformatics, Cervical cancer, Ribonucleotide reductase M2, Stromal cells, Tumor cells, Lymphocyte infiltration

## Abstract

Cervical cancer is the most common gynecologic malignant tumor, with a high incidence in 50-55-year-olds. This study aims to investigate the potential molecular mechanism of RRM2 for promoting the development of cervical cancer based on The Cancer Genome Atlas (TCGA) and the Gene Expression Omnibus (GEO). RRM2 was found to be significant upregulated in cervical tissue (*P*<0.05) by extracting the expression of RRM2 from TCGA, GSE63514, GSE7410, GSE7803 and GSE9750. Survival analysis indicated that the overall survival was significantly worse in the patients with high-expression of RRM2 (*P*<0.05). The top 1000 positively/negatively correlated genes with RRM2 by Pearson Correlation test were extracted. The gene co-expression network by Weighted Gene Co-Expression Network Analysis (WGCNA) with these genes and the clinical characteristics (lymphocyte infiltration, monocyte infiltration, necrosis, neutrophil infiltration, the number of normal/stromal/tumor cells and the number of tumor nuclei) was constructed. By screening the hub nodes from the co-expression network, results suggested that RRM2 may co-express with relevant genes to regulate the number of stromal/tumor cells and the process of lymphocyte infiltration to promote the progression of cervical cancer. RRM2 is likely to become a novel potential diagnostic and prognostic biomarker of cervical cancer and provide evidence to support the study of mechanisms for cervical cancer.

## Introduction

Cervical Cancer is one of the leading causes of cancer-related deaths in women, with an estimated 570,000 new cases and 311,000 deaths in 2018 worldwide 1. About nine out of ten (87%) deaths related to cervical cancer occurred in developing counties 2. The main risk factor for cervical cancer is human papillomavirus (HPV) infection, especially subtypes 16 (mostly associated with squamous cell carcinoma) and 18 (mostly associated with adenocarcinoma). Other predisposing factors include low socioeconomic status, early sexual activity, multiple sexual partners, immunosuppression and smoking 3. Although most patients can be treated at an early stage, poor prognosis can still be identified in patients with metastasis or recurrence 4, 5. Due to the high incidence and mortality of cervical cancer, uncovering the mechanisms of pathogenesis and identifying novel biomarkers is urgently needed.

The Ribonucleotide reductase subunit M2 (RRM2) gene is located on chromosome 2p25, a region with no copy-number variations (CNVs) or other structural alterations in human cervical cancer samples 6. RRM2, as a component of ribonucleotide reductase (RNR), plays vital roles in many critical cellular processes such as cell proliferation, invasiveness, migration, angiogenesis and senescence 7. It is frequently overexpressed and serves as an oncogene in various malignancies, including adrenocortical cancer 8, lung cancer 9, nasopharyngeal cancer 10, gastric cancer 11 and neuroblastoma 12, as well as being a predictor of poor prognosis 13-17. Our previous study had indicated that RRM2 plays a critical role in the diagnosis and treatment of cervical cancer 18. In addition, upregulation of RRM2 was found to lead to promotion of angiogenesis, whereas its downregulation significantly increased apoptosis and promoted cell cycle arrest in cervical cancer 6, 19. However, the mechanisms showing how RRM2 exerted its active role in the progression of cervical cancer were still unclear.

**Figure 1:**
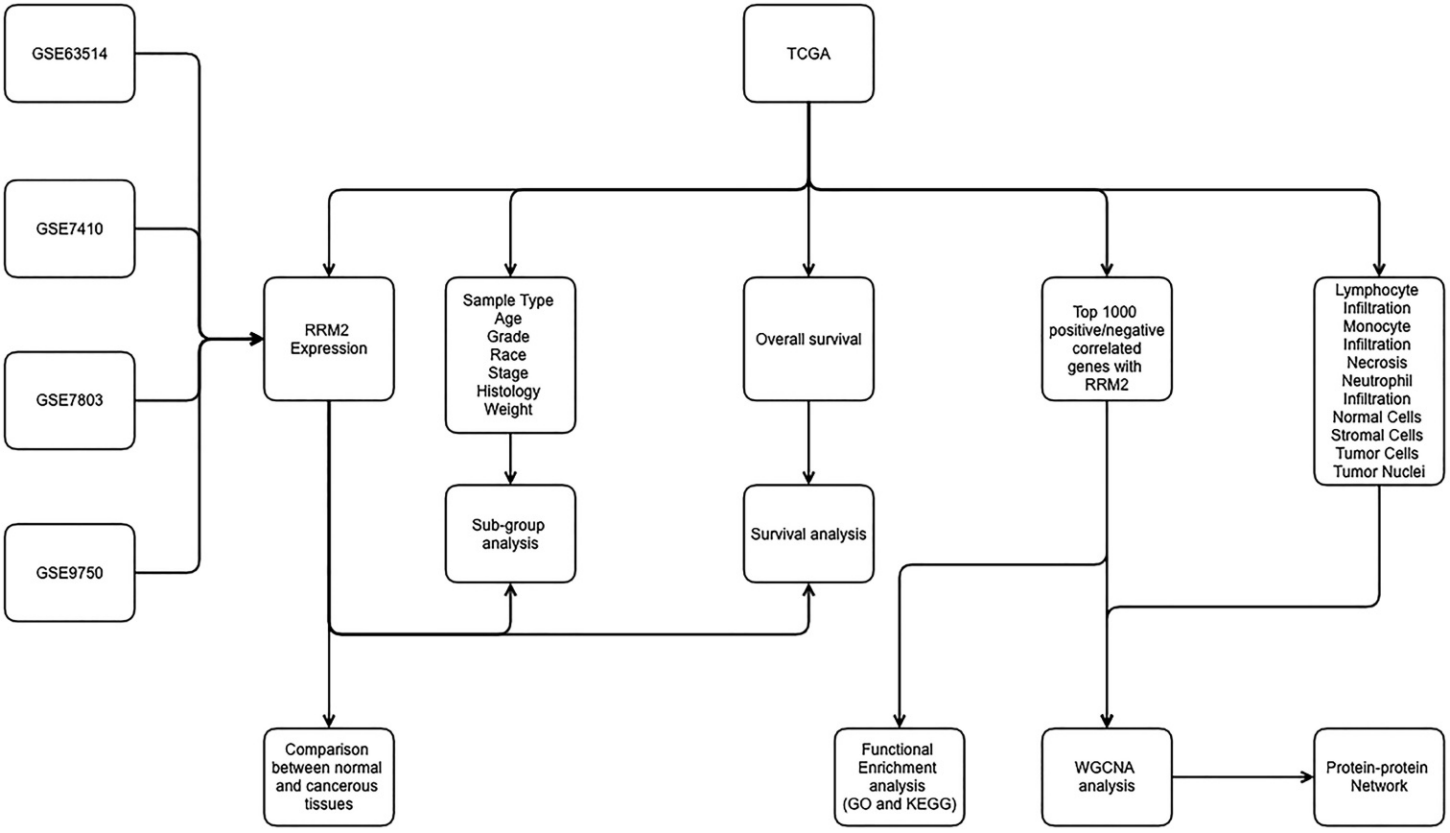
Workflow of present study for constructing gene co-expression network. TCGA, the Cancer Genome Atlas; GO, Gene Ontology; WGCNA, Weighted Gene Co-expression Network Analysis; KEGG, Kyoto Encyclopedia of Genes and Genomes.

In addition, our previous research has also shown that higher expression of RRM2 is associated with a significantly poorer overall survival 18. However, relatively little is known about the role of RRM2 in cervical cancer and even less is known of the mechanisms responsible for its effects. Based on current outstanding research, we paid special attention and interest to the potential mechanism of RRM2 in cervical cancer. Therefore, for the main purpose of revealing the potential molecular mechanism of RRM2 for promoting the development of cervical cancer, we carried out our investigation based on The Cancer Genome Atlas (TCGA) and Gene Expression Omnibus (GEO). Interestingly, we found that RRM2 may exert its promoted role in cervical cancer by reducing the number of stromal cells, increasing the number of tumor cells and promoting lymphocyte infiltration. Furthermore, our secondary objective was to determine whether RRM2 could be used as a biomarker for the prognosis of cervical cancer. In addition, the relationship between RRM2 and other biomarkers may provide more evidence for the underlying mechanisms of cervical cancer progression. The workflow of the present study is shown in **Figure 1.**

## Materials and Methods

### Raw data

The Gene Expression Omnibus (GEO; https://www.ncbi.nlm.nih.gov/geo/) is a public repository and distribution center for high-throughput microarray and sequence-based data 20. The mRNA profiles of GSE63514, GSE7410, GSE7803 and GSE9750 were downloaded from the GEO. The characteristics of these four microarrays are shown in **Table 1.** In addition, the mRNA expression datasets for cervical squamous cell carcinoma and endocervical adenocarcinoma (CESC), which contain 304 cervical cancer samples with 3 normal samples, were also downloaded from The Cancer Genome Atlas (TCGA) using cBioPortal (http://www.cbioportal.org/) 21, 22.

### Expression distribution of RRM2 in cervical cancer tissues and patients' characteristics

The expression of RRM2 was extracted from TCGA, GSE63514, GSE7410, GSE7803 and GSE9750 to identify its regulation between cervical cancer and normal tissue. If several probes were mapped to a same gene, the mean value was utilized as the final expression of this gene 23. Furthermore, the relationship between RRM2 expression and patients' characteristics was investigated through the UALCAN database, which contains TCGA level 3 RNA-seq and clinical data from 33 cancer types 24. The comparison for each clinical characteristic between each classification in RRM2 expression was performed by student-t test. Specifically, the patients' characteristics included age, tumor grade, ethnic group, stages, histological type and weight. P<0.05 was considered as significant difference. The classification of body weight was defined as follow: (1) normal weight: patients with BMI ranging from 18 to 24; (2) extreme weight: BMI ranging from 25 to 29; (3) obese: BMI ranging from 30 to 39; (4) extreme obesity: BMI equal or above 40 24.

### Overall survival analysis of RRM2 in cervical cancer

The prognostic value of RRM2 in overall survival (OS) was assessed using the CESC data in TCGA. Due to the variability in the data, we separated the patients into RRM2 high and low groups according to the best cut-off value confirmed by the pROC package based on the RRM2 expression and the survival status 25. The Cox proportional hazard model was used to compare the OS status by hazard ratio (HR) between high and low expressions of RRM2 26. The P value obtained from a log-rank test was used to indicate statistical significance of the survival correlation between groups. Additionally, P<0.05 was considered to indicate a statistically significant difference 27.

### Co-expression analysis with RRM2

To identify the potential gene co-expressed with RRM2, the LinkedOmics database was applied to extract the top 1000 significant positively/negatively correlated genes with RRM2 28. The correlation between RRM2 and each other gene was examined using the Pearson correlation test one by one. *P*<0.05 was considered to be a significant correlation.

### Functional and pathway enrichment analysis

Gene ontology (GO) and pathway analysis was carried out on the top 1000 positively/negatively co-expressed genes with RRM2 using the DAVID database (Database for Annotation, Visualization and Integrated Discovery; https://david.ncifcrf.gov/) 29. The significantly enriched biological items for biological process (BP), cellular components (CC), and molecular functions (MF), as well as pathways, were identified with *P*<0.05. The significant GO items and pathways were visualized using the ggpubr package 30.

### Weighted co-expression network construction and module selection

Weighted Gene Co-Expression Network Analysis (WGCNA) is a systems biology method for constructing relationship patterns that uses the soft threshold method to provide a more extensive and accurate correlation between transcripts compared to more general methods such as Pearson's correlation coefficient 31.

For constructing a co-expression module, we adopted the WGCNA package to analyze the relationship between the genes co-expressed with RRM2 and clinical characteristics, including lymphocyte infiltration, monocyte infiltration, necrosis, neutrophil infiltration, the number of normal/stromal/tumor cells and the number of tumor nuclei. The *hclust* function was utilized to perform sample cluster analysis on the expression data of each acquired gene in the samples and remove outlier samples. The *pickSoftThreshold* function was used to select the soft threshold. The soft threshold is a criterion based on approximate scale-free topology. The scale-free topological fitting exponential curve tends to be flat after reaching a higher value. We used the one-step method to construct the co-expression network and the *blockwiseModules* to identify co-expression modules. The minimum number of genes was set to 30 and the *plotDendroAndColors* function was applied to draw a dendrogram and to color each module for visualization.

For identifying the association between gene modules and clinical characteristics, we used gene significance (GS), defined as the correlation between genes and characteristics, to quantify the association between individual genes and the characteristics of interest. For each module, the quantitative value of module membership (MM) represented the degree of correlation between the modules and gene expression. The modules that are significant correlated with clinical characteristics by using GS and MM (*P*<0.05).

### Protein-protein interaction network analysis

After identifying the significant modules associated with clinical characteristics, the genes in each module were extracted to construct a protein-protein interaction network (PPI) and the Cytoscape software was used for visualization. To detect the hub genes that related to RRM2, the degree, closeness and betweenness value of each node in the PPI was calculated using the Centiscape plugin 32, 33. The nodes with degree, closeness and betweenness scores higher than the mean value were considered hub nodes.

## Results

### Expression distribution of RRM2 in cervical cancer tissues and patients' characteristics

**Figure 2:**
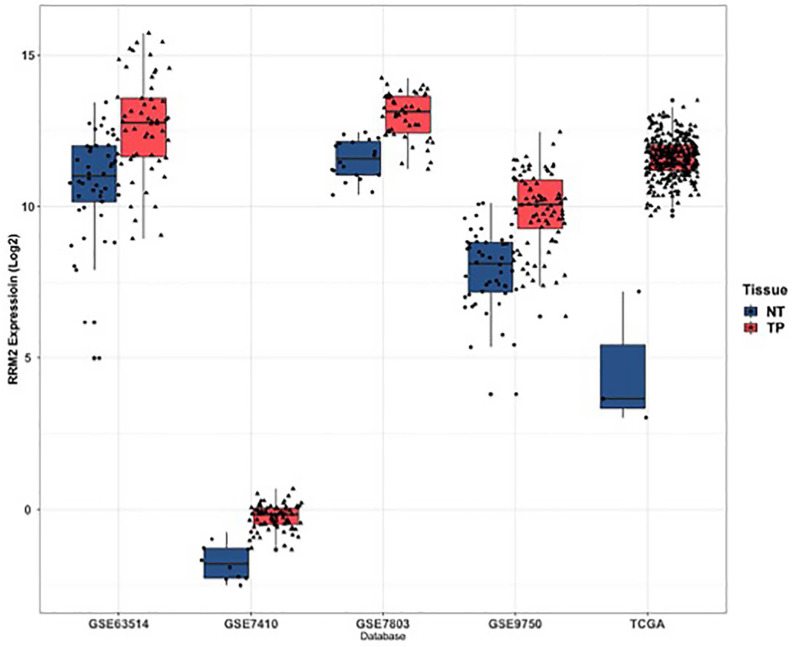
Expression of RRM2 in cervical cancer and normal tissues. RRM2 was significantly higher in cervical cancer tissues than in normal tissues (p < 0.05). NT, normal tissue; TP: primary tumor.

As shown in **Figure 2**, RRM2 was significantly upregulated in cervical cancer tissues when compared to normal tissues in TCGA, GSE63514, GSE7410, GSE7803 and GSE9750 (*P*<0.05). In terms of patients' characteristics, similar results were obtained with the expression of RRM2 significantly higher in cervical cancer patients than healthy people with respect to age, tumor grade, ethnic group, stages, histological type and weight (**Figure S1**; *P*<0.05). Specifically, the expression of RRM2 in extreme weight was higher than extreme obesity (**Figure S1F**; *P*=0.0224). However, no significant difference was detected in cervical cancer patients with respect to age, tumor grade, ethnic group, stages, or histological type (**Figure S1A-S1E**).

### Overall survival analysis of RRM2 in cervical cancer

**Figure 3:**
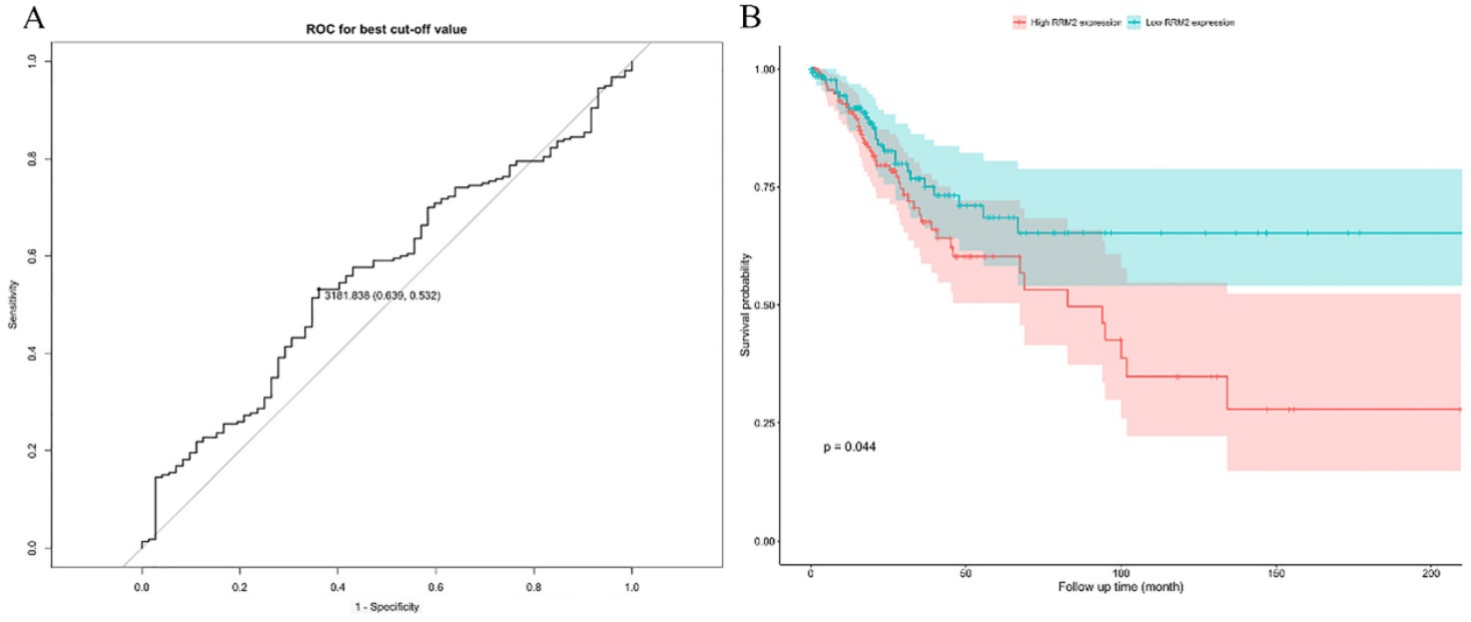
The prognostic value of differently expressed RRM2 in cervical cancer patients in survival analysis. (**A**) The optimal cutoff value is 3181.838. (**B**) The cervical cancer patients with high expression revealed a significantly poorer OS (HR: 1.621, 95%CI: 0.03834-0.9929, P value: 0.044). HR, hazard ratio; CI, confidence interval; OS, overall survival.

A log-rank test was applied to assess the OS in patients between high and low expressions of RRM2. The optimal cutoff value is 3181.838 (**Figure 3A**). The results indicated that cervical cancer patients with high expression showed a significantly poorer OS (HR: 1.621; 95% CI: 0.03834-0.9929; *P*=0.044) (**Figure 3B**).

### Co-expression analysis with RRM2

**Figure 4:**
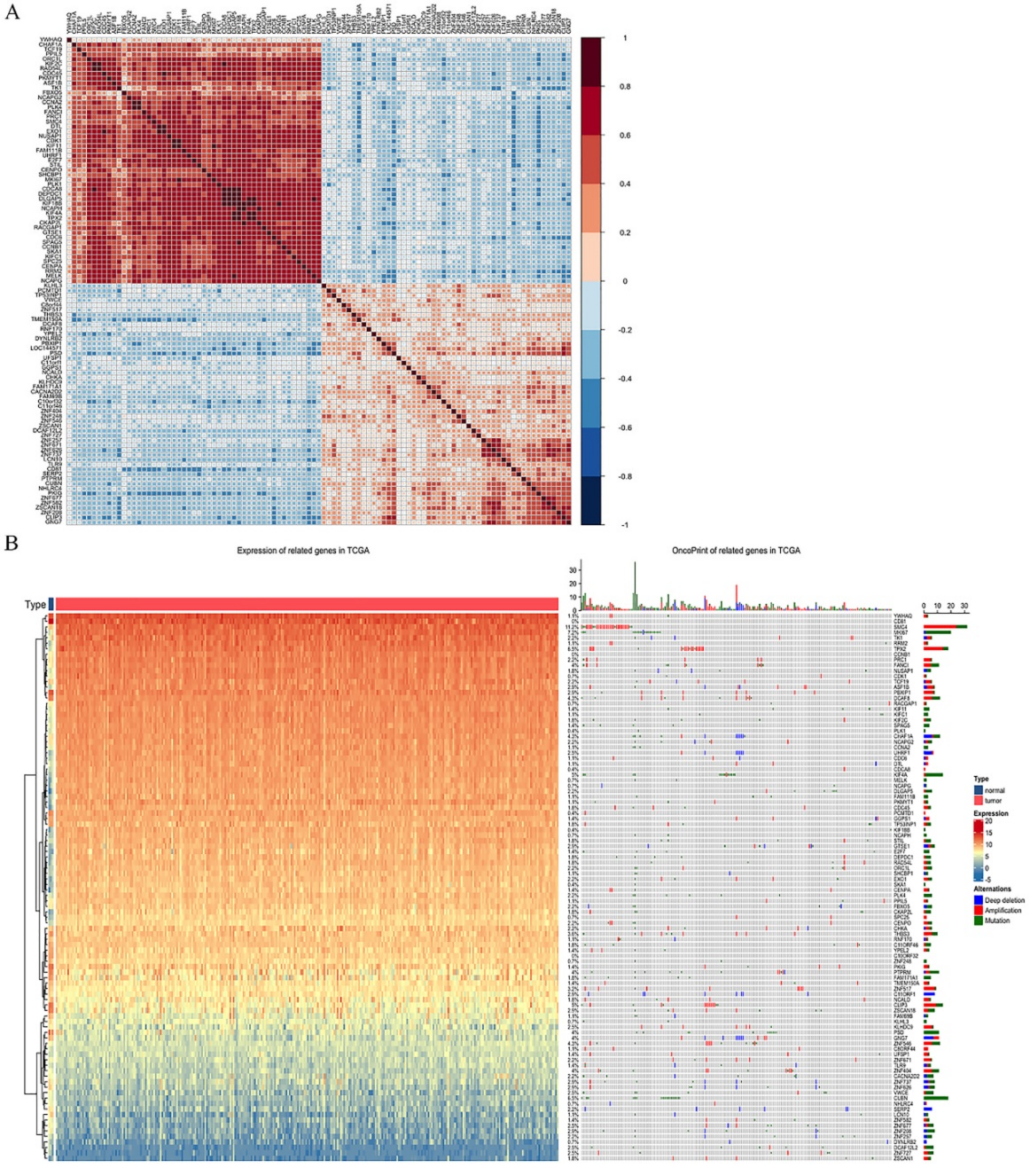
Co-expression analysis with RRM2. (**A**) Correlations between the top 50 positively/negatively correlated genes. Red represents positive correlation, and blue indicates negative correlation. (**B**) Expression between normal and cervical cancer tissue and oncoprint of the top 50 positively/negatively correlated genes in TCGA.

By extracting the top 1000 significant positively/negatively correlated genes with RRM2, a total of 2000 potential genes were acquired. **Table 2 and 3** shows the top ten significant positively/negatively correlated genes. Of which, TCF19 (R = 0.539, *P* = 2.51*10^-24^), CDCA8 (R = 0.518, *P* = 2.53*10^-22^) and CENPO (R = 0.508, *P* = 4.63*10^-21^) were the top three positively correlated genes with RRM2, whereas PCMTD1 (R = -0.374, *P* = 1.69*10^-11^), TP53INP1 (R = -0.362, *P* = 7.19*10^-11^) and ZNF582 (R = -0.358, *P* = 1.32*10^-10^) represented the top three negatively correlated genes. **Figure 4A** shows the correlation matrix for the top 50 significant positively/negatively correlated genes, and **Figure 4B** shows their expression and oncoprint in TCGA.

### Functional and pathway enrichment analysis

**Figure 5:**
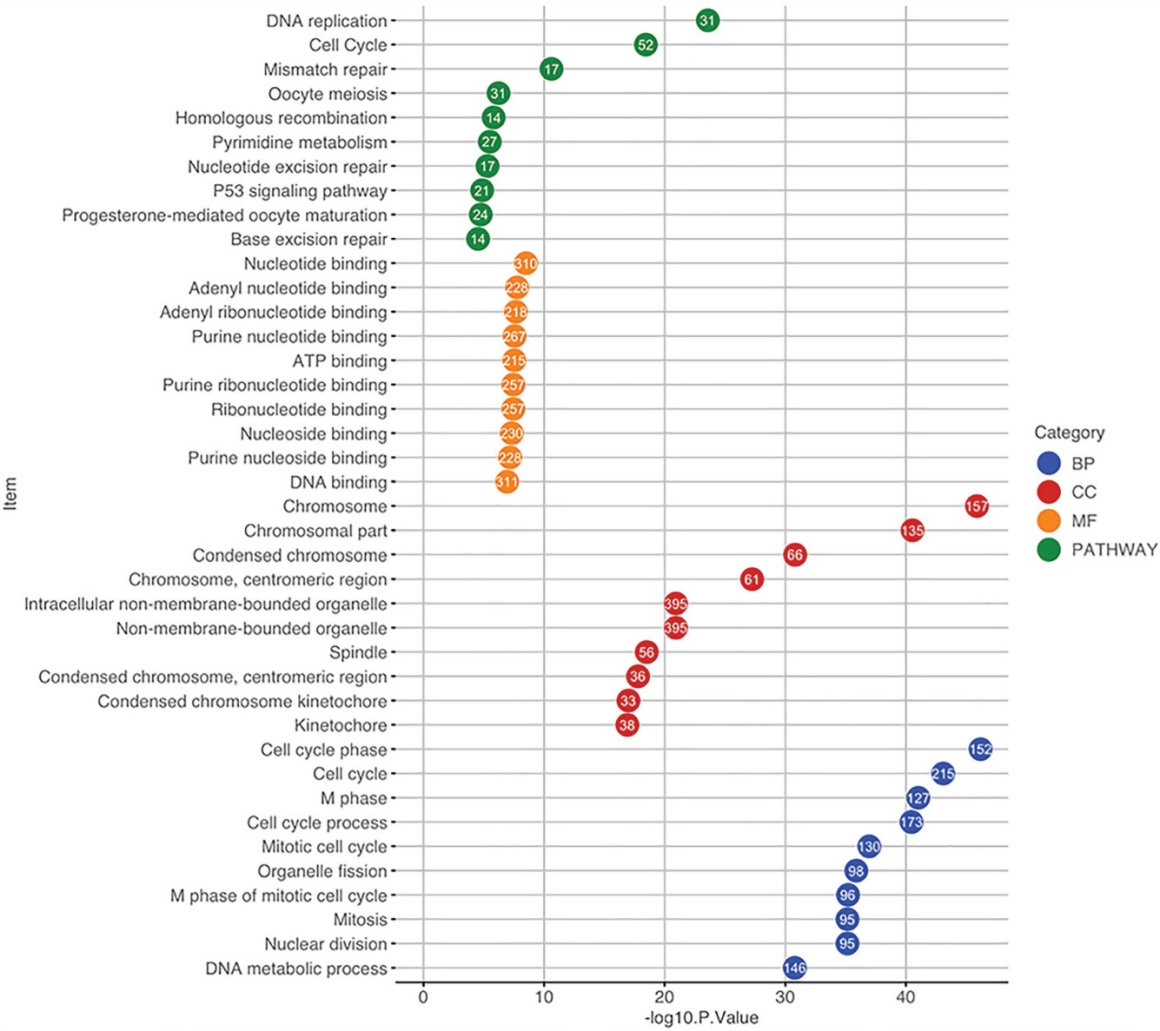
Functional and pathway enrichment analysis using the DAVID database. The GO items and pathways were differentiated by colors. The position of a circle corresponds to the enrichment significance of each term and the number in the circle indicates the enriched gene count. GO, Gene Ontology; CC, cellular component; BP, biological process; MF, molecular function.

The 2000 significant correlated genes were used for GO and pathway enrichment analysis using the DAVID database. For GO analysis, when considering BP, these genes were mainly enriched in *cell cycle*, *cell cycle phase* and *M phase*. With regards to CC, the top three enriched items were *chromosome*, *chromosomal part* and *centromeric region*. In terms of MF, *nucleotide binding*, *adenyl nucleotide binding* and *adenyl ribonucleotide binding* were in the first three enriched places. Pathway enrichment analysis indicated that the top three significantly enriched pathways were *DNA replication*, *cell cycle* and *mismatch repair* (**Figure 5**).

### Weighted co-expression network construction and module selection

Using the expression data of the 2000 significant correlated genes in 307 samples in CESC, the WGCNA algorithm was used to generate the co-expression module. The *hclust* function was used to perform sample clustering analysis, and no outlier samples were found (**Figure S2A**). As topological analysis was required to construct the network, the soft threshold power value was set to 5 according to the scale-free network standard (**Figure S2B**). According to the determined power value, six effective modules with gene number more than 30 were detected with one gray module containing genes that cannot be clustered as a module (**Figure S3**).

**Figure 6:**
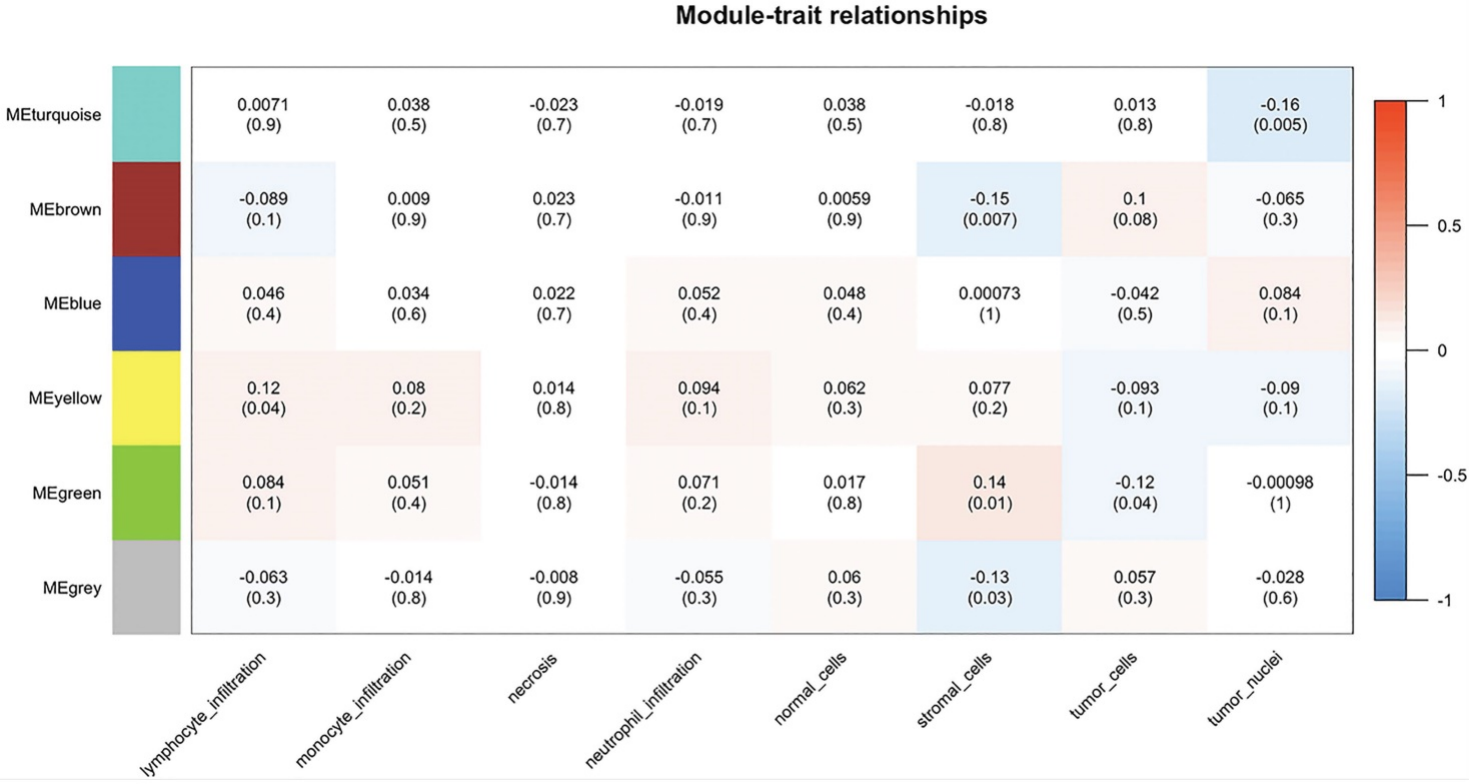
Module-patient trait associations. The relationship between modules and patient traits was analyzed using WGCNA. Each row corresponds to a module eigengene, each column to a trait. Each cell contains the corresponding correlation and *p*-value. The table is color-coded by correlation according to the color legend. WGCNA, Weighted Gene Co-expression Network Analysis; ME, module eigengene.

By importing the clinical characteristics including lymphocyte infiltration, monocyte infiltration, necrosis, neutrophil infiltration, the number of normal/stromal/tumor cells and the number of tumor nuclei, module trait association analysis was performed to identify modules that were significantly related to clinical traits. The yellow module was found to positively correlate with lymphocyte infiltration (R = 0.12, *P* = 0.04), whereas the brown module was negatively correlated with the number of stromal cells (R = -0.15, *P* = 0.007). In addition, the green module was negatively correlated with the number of stromal cells (R = 0.14, *P* = 0.01) and the number of tumor cells (R = -0.12, *P* = 0.04), and the turquoise obtained similar results for the number of tumor nuclei (R = -0.16, *P* = 0.005) (**Figure 6**).

### Protein-protein interaction network analysis

**Figure 7:**
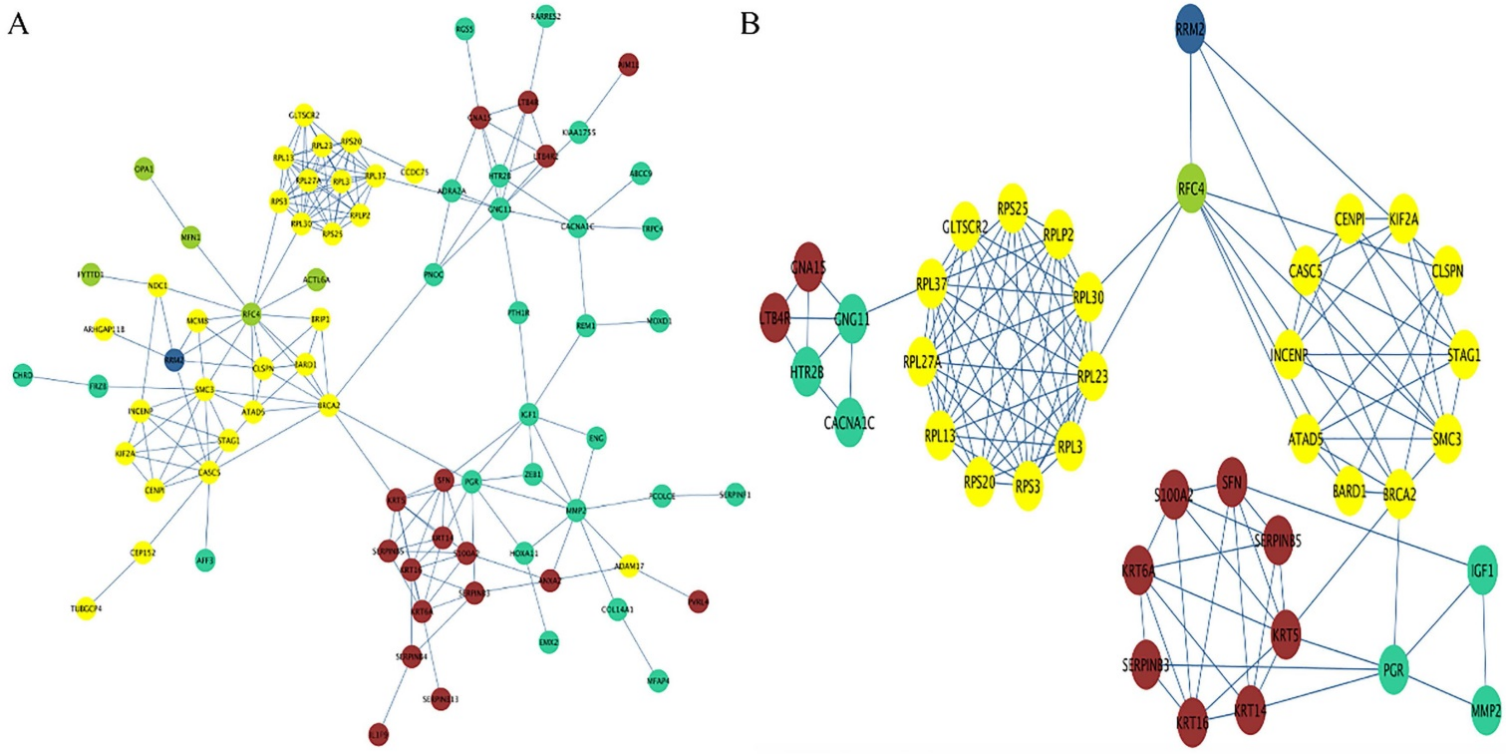
PPI network. (**A**) The preliminary PPI network of these genes in each identified module. The color of each node represents its corresponding module. (**B**) The subnetwork containing 39 nodes and 125 edges was extracted based on the criteria of degree, closeness and betweenness scores of nodes being higher than the mean value. RRM2 may co-express with RFC44 to increase the number of tumor cells and reduce the number of stromal cells, and subsequently regulate the expression of the RPL family (RPL30, RPL23 and RPL3 et al.) or CENPI, KIF2A and CASCS to promote lymphocyte infiltration. After the lymphocyte infiltration, CNG11 or PGR would be regulated to enhance the malignant behavior by increasing the number of tumor nuclei, and further regulate the expression of GNA15/LTB4R or SFN/KRT14 to reduce the number of stromal cells. In another route, RRM2 may also directly interact with the genes in the yellow module to exert a promoted effect on it. Circles represent genes; lines represent interactions between these selected genes. PPI, protein-protein interaction.

By extracting the genes in each identified module, a preliminary PPI with 79 nodes and 190 edges was constructed, with the color of each node representing their corresponding module (**Figure 7A**). Using degree, closeness and betweenness scores of nodes higher than the mean value, a subnetwork containing 39 nodes and 125 edges was extracted (**Figure 7B**). Combining the subnetwork with the result of module trait association analysis, the possible mechanisms for RRM2 to promote the progression of cervical cancer formed as follows. RRM2 may co-express with RFC44 (correlated with stromal cells and tumor cells) to increase the number of tumor cells and reduce the number of stromal cells, and subsequently regulate the expression of the RPL family (RPL30, RPL23 and RPL3 et al.) or CENPI, KIF2A and CASCS (correlated with lymphocyte infiltration) to promote lymphocyte infiltration. After the lymphocyte infiltration, the expression of CNG11 or PGR (correlated with tumor nuclei) would be regulated to enhance the malignant behavior by increasing the number of tumor nuclei, and further regulate the expression of GNA15/LTB4R or SFN/KRT14 (correlated with stromal cells) to reduce the number of stromal cells. In another route, RRM2 may also directly interact with the genes in the yellow module to exert a promoted effect on it. These processes may be structured as a vicious circle.

## Discussion

Cervical cancer is the third most commonly diagnosed gynecological malignant tumor and the fourth major cause of cancer‐related death, with especially high morbidity in developing countries 34. Although the etiological role of HPV in the development of cervical cancer has been confirmed 35, the specific molecular mechanism remains unclear. Previous study had revealed that RRM2 was a downstream target for the HPV E7 gene, and that HPV E7 can induce upregulation of RRM2 and then promotes cervical carcinogenesis via ROS-ERK1/2-HIF-1α-VEGF-induced angiogenesis 6. To date, RRM2 had been reported as having an important impact on cervical cancer 18, 36, 37. Furthermore, several non-coding RNA, such as long non-coding RNA (LncRNA) or micro RNA (miRNA), regulate the occurrence and development of cervical cancer through RRM2 38, 39. However, the mechanisms showing how RRM2 promote cervical cancer are still unclear. In the present study, we investigated the promotion role of RRM2 for cervical cancer using WGCNA.

Our results indicated that RRM2 is upregulated and significantly associated with poor prognosis in cervical cancer, and these results are in line with previous studies 18, 19, 36, 40. With 192 samples (29 normal, 30 low-grade dysplasia, 30 high-grade dysplasia and 103 invasive cancer tissue specimens) and SiHa cervical cancer cells, Su et al. reported the expression of RRM2 in cancer tissues was increased when compared to high-grade dysplasia, low-grade dysplasia or normal cervical tissues 40. Furthermore, higher expression of RRM2 was significantly correlated with worse survival. Wang et al. demonstrated that the RNA levels of RRM2 in cervical cancer tissues were significantly higher than paracarcinoma tissues by analyzing 38 pairs of cervical cancer and normal cervical tissues 19. They also found that downregulated RRM2 promoted apoptosis and cell cycle arrest, as well as inhibiting tumor formation. In addition, bioinformatics analyses also indicated that OS was significantly better in the low-expression RRM2 group 18, 36.

The results of the present study found that RRM2 may exert its promoted effect in cervical cancer by reducing the number of stromal cells, increasing the number of tumor cells and promoting lymphocyte infiltration. At present, the study of mechanisms for RRM2 in cervical cancer is limited. Su et al. reported that RRM2 upregulation was significantly correlated with deep stromal invasion and parametrial invasion 40. Furthermore, stromal invasion is considered as a significant predictive factor of OS for cervical cancer 41-43. In other malignancies, Sun et al. reported that RRM2 was a positive regulator of glioma progression and it contributed to the migration and proliferation of glioma cells 44. Zhang et al. found that overexpression of RRM2 promoted the growth of tumor cells, as well as positively affecting the overall angiogenic activity of tumor cells, in oropharyngeal carcinoma 45. Li et al. presented similar results that increased expression of RRM2 is essential for cell proliferation in glioblastoma, while knockdown of RRM2 led to cancer cells arrested at the G1 phase and promoted apoptosis to inhibit cell growth 46. Unfortunately, the study of the relationship between RRM2 and lymphocyte infiltration is also limited. Lu et al. reported that RRM2 may play an important role in the infiltration and metastasis of colorectal cancer by analyzing 56 pairs of cancerous and normal tissues 47. Nevertheless, RRM2 still had potential association with the regulation of the stromal cells, tumor cells and lymphocyte infiltration.

In addition, there are some genes and proteins that have been associated with the effects and mechanisms of RRM2 in cervical cancer progression. As a tumor inhibitor, p53 plays a pivotal role in cervical cancer 48, He et al. indicate that both RRM1 and RRM2 were positively controlled by mTOR signaling while suppressed by p53 signaling 49. The HPV E7 protein targets and inactivates the retinoblastoma protein (Rb), resulting in the release of active E2F and the activation of E2F target genes 50. Research found that RRM2 was a novel downstream target activated by HPV E7, and promoted angiogenesis by producing ROS, activating the ERK1/2 signaling pathway and inducing expression of HIF-1a and VEGF, leading to cervical angiogenesis and carcinogenesis 6. At present, evidence of the relationship between RRM2 and CDKN3 in cervical cancer is limited, but their relationship can be found in other cancers, such as hepatocellular carcinoma, breast cancer, renal carcinoma and lung cancer 51-54. Qi et al. have found RRM2 and CDKN3 may become biomarkers for diagnosis and poor prognosis of breast cancer 51. Zhou et al. have also found there is a correlation between RRM2 and CDKN3 in hepatocellular carcinoma 52. Wei et al. found RRM2 and CDKN3 associated with the metastasis of Clear cell renal cell carcinoma 53. MacDermed et al. have also found their relationship in lung adenocarcinoma 54. Therefore, we speculate that there is an interaction between RRM2 and CDKN3 in cervical cancer, which needs to be confirmed by further study 55.

Our studies also had limitations. First, we selected the top 1000 positively/negatively correlated genes with RRM2 for WGCNA, and so the results may change if we enlarge the number of selected genes. Second, the hub nodes were identified based on the topological parameters such as degree, closeness and betweenness. Applying another algorithm may also lead to the alteration of the results. Third, as the present study is an *in-silico* study, the results need to be further validated.

## Conclusions

In conclusion, the expression of RRM2 is upregulated in cervical cancer and promotes cancerous progression. RRM2 may exert its promoted role in cervical cancer by reducing the number of stromal cells, increasing the number of tumor cells and promoting lymphocyte infiltration. It is likely to become a novel potential diagnostic and prognostic biomarker of cervical cancer.

## Supplementary Material

Supplementary figures.

## Figures and Tables

**Table 1:** Basic characteristics of the four microarray datasets from GEO

Profile	RNA type	Platform	Organism	Sample size (T/N)	Published Year	Country
GSE63514	mRNA	GPL570	Homo sapiens	28/24	2015	USA
GSE7410	mRNA	GPL1708	Homo sapiens	40/5	2008	Netherlands
GSE7803	mRNA	GPL96	Homo sapiens	3/10	2007	USA
GSE9750	mRNA	GPL96	Homo sapiens	42/24	2008	USA

T: Tumor; N: Normal; GEO: Gene Expression Omnibus.

**Table 2:** Top 10 positively correlated genes with RRM2

Gene	Cor.	*P* value	Sample size
TCF19	0.539	2.51e-24	304
CDCA8	0.519	2.53e-22	304
CENPO	0.505	4.63e-21	304
CCNA2	0.496	2.68e-20	304
KIF2C	0.493	4.81e-20	304
KIF4A	0.491	8.28e-20	304
RACGAP1	0.488	1.41e-19	304
ORC1L	0.486	1.87e-19	304
KIFC1	0.481	4.77e-19	304
MELK	0.474	1.97e-18	304

Cor.: Correlation,

**Table 3:** Top 10 negatively correlated genes with RRM2

Gene	Cor.	P value	Sample size
PCMTD1	-0.374	1.69e-11	304
TP53INP1	-0.362	7.19e-11	304
ZNF582	-0.358	1.32e-10	304
ZNF737	-0.356	1.55e-10	304
TMEM150A	-0.345	6.20e-10	304
ZNF248	-0.343	7.99e-10	304
LOC144571	-0.339	1.31e-09	304
FAM171A1	-0.337	1.57e-09	304
ZNF626	-0.335	2.05e-09	304
RNF170	-0.334	2.39e-09	304

Cor.: Correlation.
