# Supplementary material for: Potential mechanism of RRM2 for promoting Cervical Cancer based on weighted gene co-expression network analysis
Source: Int J Med Sci. 2020 Aug 29;17(15):2362–72. doi: 10.7150/ijms.47356 (PMC7484645; doi:10.7150/ijms.47356)

Supplementary materials

Figure S1 RRM2 expression in cervical cancer patients stratified by age, tumor grade, ethnic group, stages, histological type and weight. $P<0.05$ was considered as significant difference. (A) RRM2 expression distribution for age ($P>0.05$); (B) RRM2 expression distribution for grade ($P>0.05$); (C) RRM2 expression distribution for ethnic group ($P>0.05$); (D) RRM2 expression distribution for stage ($P>0.05$); (E) RRM2 expression distribution for histology ($P>0.05$); (F) RRM2 expression distribution for weight based on BMI (Body Mass Index). The expression of RRM 2 in extreme weight was higher than extreme obesity ($P = 0.0224$).

Figure S2 Sample clustering analysis and soft-thresholding values (β) estimation. (A) Sample clustering to detect outliers indicated that no outliers were present that required removal from the subsequent analysis. Branches of the dendrogram represent together hub genes that are correlated positively. (B) The optimal soft-thresholding (β) was determined as 5. The left panel displays the influence of soft-thresholding power (x-axis) on the scale-free fit index (y-axis). The right panel shows the influence of soft-thresholding power (x-axis) on the mean connectivity (degree, y-axis).

Figure S3 Clustering dendrograms of genes, with dissimilarity based on topological overlap, together with assigned module colors. Additionally, the clustering diagram and six effective modules with gene number more than 30 and screening based on gene expression pattern are presented. WGCNA, Weighted Gene Co-Expression Network Analysis.

Figure S1

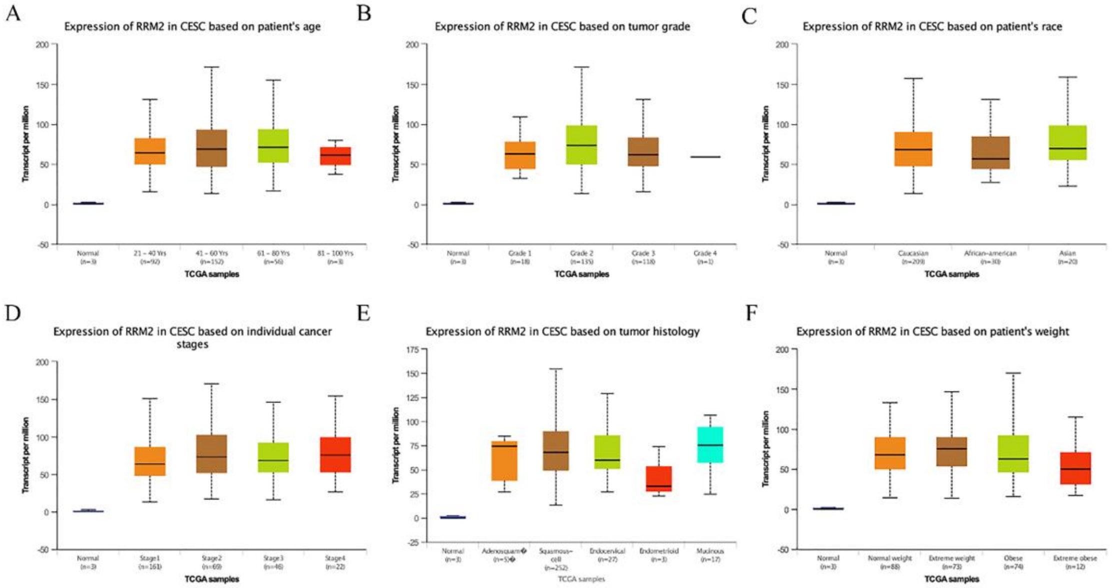

Figure S2

A

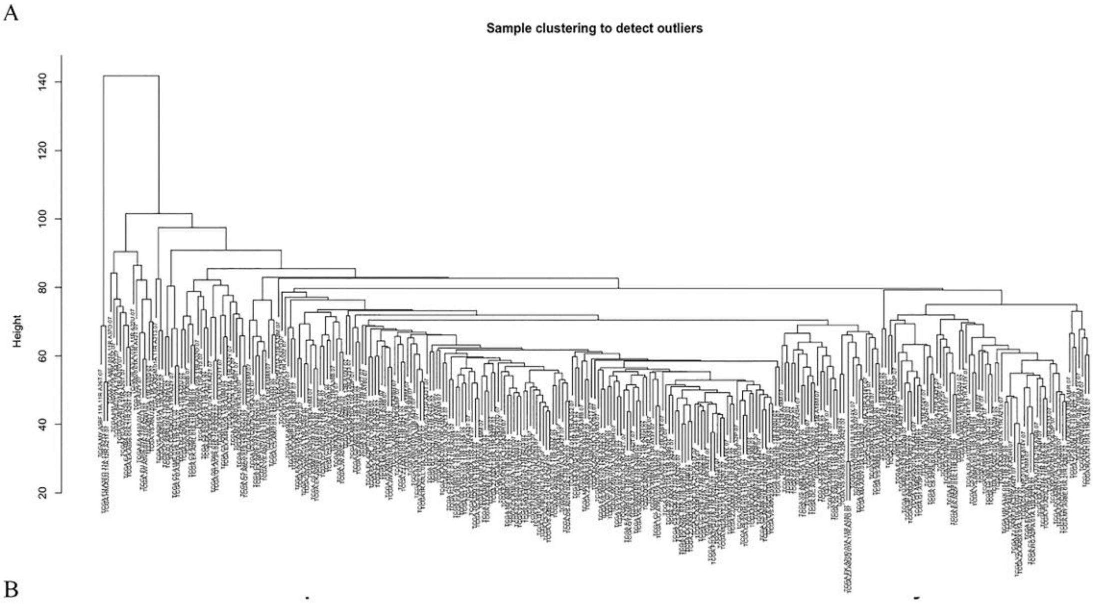
Sample clustering to detect outliers
Height
20
40
60
80
100
120
140

B

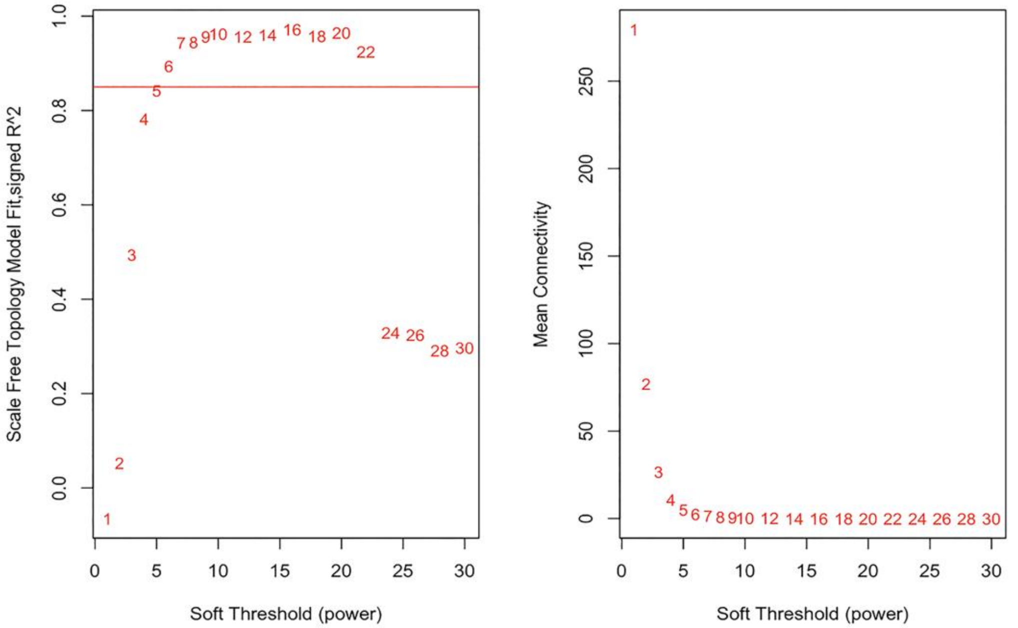
Scale Free Topology Model Fit,signed R^2
Soft Threshold (power)
Mean Connectivity
Soft Threshold (power)

Figure S3

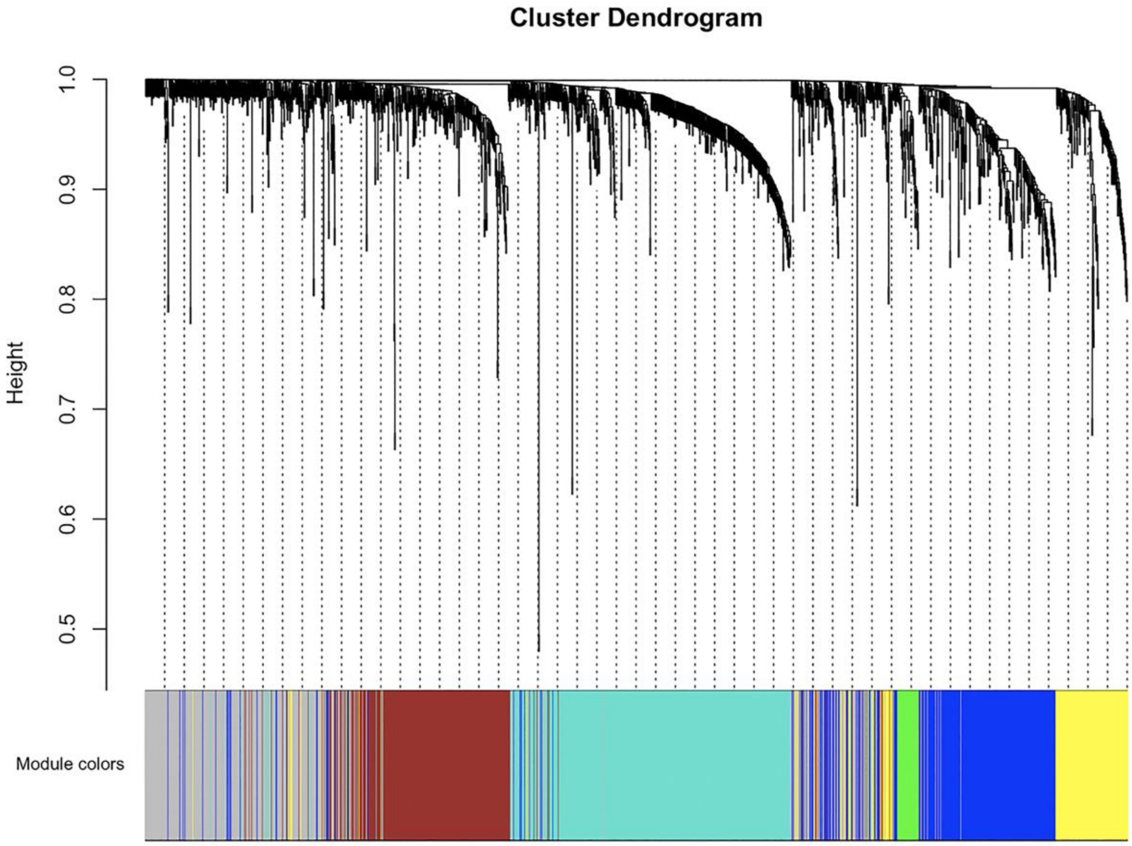

Supplement: Supplementary file 1 — Supplementary figures. [file ijmsv17p2362s1.pdf]
